# A theoretical review of the Proteus effect: understanding the underlying processes

**DOI:** 10.3389/fpsyg.2024.1379599

**Published:** 2024-06-26

**Authors:** Anna Martin Coesel, Beatrice Biancardi, Stéphanie Buisine

**Affiliations:** CESI LINEACT, Nanterre, France

**Keywords:** Proteus effect, avatar embodiment, virtual reality, self-perception theory, deindividuation, priming, virtual environments

## Abstract

Humans' inherent fascination for stories can be observed throughout most of our documented history. If, for a long time, narratives were told through paintings, songs, or literature, recent technological advances such as immersive virtual reality have made it possible for us to interact with storylines and characters in a completely new manner. With these new technologies came the need to study how people interact with them and how they affect their users. Notably, research in this area has revealed that users of virtual environments tend to display behaviors/attitudes that are congruent with the appearance of the avatars they embody; a phenomenon termed the Proteus effect. Since its introduction in the literature, many studies have demonstrated the Proteus effect in various contexts, attesting to the robustness of the effect. However, beyond the first articles on the subject, very few studies have sought to investigate the social, affective, and cognitive mechanisms underlying the effect. Furthermore, the current literature appears somewhat disjointed with different schools of thought, using different methodologies, contributing to this research topic. Therefore, this work aims to give an overview of the current state of the literature and its shortcomings. It also presents a critical analysis of multiple theoretical frameworks that may help explain the Proteus effect. Notably, this work challenges the use of self-perception theory to explain the Proteus effect and considers other approaches from social psychology. Finally, we present new perspectives for upcoming research that seeks to investigate the effect of avatars on user behavior. All in all, this work aims to bring more clarity to an increasingly popular research subject and, more generally, to contribute to a better understanding of the interactions between humans and virtual environments.

## 1 Introduction

For most of our recent history, it seems that humans have had a fascination for stories. This inclination can be observed across cultures and millennia alike. From Paleolithic cave paintings to centuries of literature or today's immense media landscape, stories seem to be ever-present around us. Even more fascinating is our ability to be transported into a narrative in such a way that we forget, for a moment, its fictitious nature (i.e., narrative transportation, Green et al., 2004). Some scholars have theorized that this inclination could stem from our deeply social nature as humans (Oatley, 1999). Our appeal for storytelling, characters, and plotlines could therefore stem from how important understanding social dynamics around us is since it is a key component of our survival as a social species. Thus, stories can be thought of as a way to simulate or recount critical scenarios without suffering the possible consequences of these situations. In many ways, this disposition could represent an evolutionary advantage as it trains our social and emotional intelligence and helps to communicate societal norms.

If books, songs, or paintings have been a way to tell stories for centuries, today's technologies offer new revolutionary ways to interact with narratives and characters. Notably, immersive virtual reality (IVR) is a particularly interesting way to do so. Through this new technology, we are able, for the first time, to look through the eyes of an avatar completely different from us. Even more remarkable is the way our brains seem to adopt the new virtual reality while consciously knowing that what is shown is simulated by a computer. For example, a study has been able to demonstrate that people show genuine fear when their virtual body might be in physical danger (Argelaguet et al., 2016). Another one showed that people seem to adapt their behavior when extra limbs are added to their virtual bodies (Arai et al., 2022).

These phenomena illustrate humans' inherent tendency to accept and engage in realities we know to be fictitious. Faced with the democratization of immersive technologies and the resulting new forms of interaction, researchers have sought to investigate the effects that embodying different virtual bodies would have on the users of virtual environments. Specifically, embodying avatars with physical characteristics that may evoke strong stereotypical behaviors or attitudes has been the subject of multiple studies. So, could embodying a Nobel prizewinner, an elderly person or even God change how people behave? If so, through which mechanisms? These questions are at the center of what researchers have termed the Proteus effect, and the subject of this review.

The goal of this article is to provide a critical review of the theoretical frameworks that could explain the Proteus effect. To our knowledge, no other review about the Proteus effect has addressed this question as extensively. The review is organized as follows. In Section 2, we summarize the current state of literature surrounding the Proteus effect, which brings us to question which processes could be responsible for this phenomenon. In Section 3, we review the hypotheses that have already been addressed in previous research, and thereafter, in Section 4 we address new or insufficiently researched hypotheses. In Section 5, we will present which hypotheses we find to be the most relevant to explain the Proteus effect. We conclude in Section 6 and give recommendations for future research on the Proteus effect.

## 2 Proteus effect

In Greek mythology, the sea God Proteus is believed to be the first son of the Olympian God of the sea, Poseidon. Proteus is described by Homer in the Odyssey as “the old man of the sea” and the herdsman of sea monsters and animals (Homer, 1919). Alongside knowing everything past, present, and future, Proteus also had the ability to change his shape at will. This last feature is thought to reflect the ever-changing quality of the sea from which the God originated. It was noted, however, that Proteus did not wish to share his extraordinary knowledge with others. Notably, he was known to change his shape to escape from the humans who wished to use his prophetic abilities. Since, the Greek God has given its name to several concepts, notably the English adjective “protean” that describes one's tendency to change frequently.

### 2.1 Princeps publication

In 2007, two researchers at Stanford University published a paper detailing two experiments in which participants embodied an avatar within an immersive virtual environment (Yee and Bailenson, 2007). Through those experiments, the researchers sought to investigate how the physical characteristics of the avatars could change the way users behave while embodying them. In their first experiment, participants were assigned avatars that could be either attractive, neutral, or unattractive. They then started the task by observing their avatar in a virtual mirror for about a minute. After that, participants were asked to interact with a confederate inside the virtual environment. The results of this first experiment showed that, compared to participants using less attractive avatars, users embodying an attractive avatar tended to come physically closer to the confederate, but also reveal more information about themselves (self-disclosure).

In the second experiment, the researchers manipulated the height of the avatars so that participants would either be shorter, taller, or the same height as the confederate. In this experiment, participants took part in a negotiation task in which they took turns splitting a sum of money with the confederate. The other person could then accept the split and take the money or refuse, in which case none of them would receive any money. The results of the negotiation task showed that users embodying a taller avatar tended to propose more unfair splits (to their advantage) compared to those using a shorter avatar. On the other hand, participants embodying shorter avatars were more likely to accept offers that did not benefit them.

Importantly, in both experiments, the confederates were blind to attractiveness and height manipulations (i.e., for all experimental conditions, they perceived avatars as either neutrally attractive or the same height as them). Thus, the observed effects on the participants cannot be explained by a change in the behavior of the confederate in response to those manipulations (i.e., behavioral confirmation). From these results, the authors concluded that participants' behavior changed in response to the appearance of their avatars. Specifically, it seemed that participants tended to display behaviors that were congruent with the features of their virtual representations. Indeed, we know from the literature that people commonly believe attractive individuals to be more confident (Dion et al., 1972), and taller people to be more likely to be leaders (Hamstra, 2014). These commonly held stereotypes could explain why participants with attractive and tall avatars acted more self-confident and self-serving in the tasks.

Based on these results, Yee and Bailenson (2007) defined the Proteus effect as the process by which users conform their behavior to the expectations evoked by the physical appearance of their avatars. In other words, users embodying an avatar seem to exhibit behaviors that an outside observer would consider consistent with the appearance of their avatars.

### 2.2 Further demonstrations

Since this first publication, many studies have replicated the Proteus effect using a wide variety of avatars and contexts. Within this literature, the avatar features manipulated by the researchers vary greatly. In this Section, we describe the main characteristics that have been used to show the Proteus effect in past research. These will be presented in two different categories: bodily features, which encompass traits such as gender, race, or attractiveness, and characters, which include the reference or use of famous individuals, uniforms, or even non-human figures.

Within the bodily features category, one of the aspects that is often manipulated is the gender of the embodied avatar (Yee et al., 2011; Ratan and Dawson, 2016). In one study, this manipulation was used to demonstrate stereotype threat (i.e., conforming to a negative stereotype about oneself) among participants completing a mathematics test while embodying customized avatars (Ratan and Sah, 2015). Results showed that participants embodying a female avatar had worse scores on the test compared to the participants embodying male avatars. The authors concluded that participants conformed to the negative sexist stereotype associated with the gender of their avatar (i.e., women are bad at mathematics). Other studies manipulating gender have shown differences in pro-social behaviors within online games depending on the gender of the avatar used (Yee et al., 2011). However, several studies have also failed to show behavioral differences using avatars of different genders (Chang, 2014; Kaye et al., 2018). These shortcomings could be explained by the fact that individuals tend to have quite complex attitudes toward gender and the associated stereotypes. These attitudes may interact with many other factors that are not accounted for in these experimental protocols. This could, therefore, prevent participants from displaying the stereotypical behaviors expected from them.

Physical features of the avatars manipulated in other experiments include height (Yee and Bailenson, 2007; Yee et al., 2009), attractiveness (Yee and Bailenson, 2009; Yulong et al., 2015), age (Reinhard et al., 2020), ethnicity (Ash, 2016), or weight (Pea et al., 2016; Joo and Kim, 2017; Ferrer-García et al., 2018). For example, researchers showed that, after having cycled for a period of time, participants embodying avatars considered to be less athletic tended to report higher levels of perceived exertion compared to participants embodying more athletic avatars. Moreover, these avatars also influenced users' heart rate such that less athletic looking avatars were associated to higher heart rate during cycling compared to athletic avatars. Another study showed a decrease in participants' walking speed post-VR use when embodying an elderly avatar compared to those using a younger avatar (Reinhard et al., 2020). Finally, the category of bodily features also includes studies testing the effect of structural changes to the avatar's body (Won et al., 2015). This has been done using avatars with an abnormal number of limbs such as six fingers (Hoyet et al., 2016), or avatars with tails (Steptoe et al., 2013).

It should be noted that using negative stereotypes relating to race, gender, or body size has been criticized for its lack of consideration of the sensitive nature of these topics (Clark, 2020). Using avatars to highlight the existence of harmful stereotypes is not inconsiderate in itself. Rather, the criticism can stem from a lack of acknowledgment that these stereotypes have very detrimental consequences for a very large part of the population and that the ultimate goal should be to find ways to minimize them.

The second category of features manipulated within the Proteus effect literature, termed “characters”, includes studies in which participants embody well-known characters or figures that evoke strong attributes. One way this is done is through the embodiment of famous individuals associated with extraordinary qualities. For example, one such study showed an increase in performance on a cognitive task when using an avatar resembling Albert Einstein (Banakou et al., 2018). Another experiment has shown increased divergent thinking abilities, a component of creative thinking, when embodying an avatar of Leonardo da Vinci (Gorisse et al., 2023).

Another way in which researchers have used characters to study the Proteus effect is through the use of uniforms (Guegan et al., 2016, 2017). Peña et al. (2009) for example showed a link between the use of avatars dressed in either black cloaks or Ku Klux Klan (KKK) uniform and aggression. In another study, Buisine et al. (2016) used two different types of avatars in their experiment: either ones representing typical users of public transport or ones that were reminiscent of the stereotypical image of an inventor. The distinction between the two groups was communicated through the avatars' clothes (e.g., jeans and tee-shits vs a laboratory coat). In this experiment, engineers were asked to come up with innovative applications of a new technology in public transport. Results showed that the ones embodying the inventor avatars found more technological-centered ideas whereas the ones embodying user avatars were more likely to propose user-centered ideas. These results showed how the appearance of the avatars, and more specifically their clothing, shaped the creative thinking of engineers.

The last mention in this category concerns the embodiment of non-human figures, which may convey traits that humans cannot possess (Ahn et al., 2016). One experiment found a decrease in physiological responses to danger when participants embodied an avatar resembling the Christian God (Frisanco et al., 2022). In another study, participants were found to be more efficient during a task in which they had to block incoming projectiles when they used alien-like avatars, compared to human-like avatars (Christou and Michael-Grigoriou, 2014). These results indicate a tendency for users to feel less physically vulnerable and more confident when embodying figures with supernatural attributes.

Nonetheless, if avatar appearance has been shown to impact users of virtual environments it is also important to note that other elements surrounding such experiences can also affect how avatars are perceived. Indeed, factors such as avatar-environment congruence (Mal et al., 2023), from which point of view the user sees the avatar (i.e., first or third) (Gorisse et al., 2017), or the type of displays being used (Hepperle et al., 2022) have been found to affect the users' perception of avatars. Beyond avatars, other core aspects of virtual environments such as the level of detail of the graphics or the context cues provided to the users also impact user behavior (for a review see Neo et al., 2021). Even if these elements are not the subject of this review, it is important to consider them when trying to understand avatar and user interactions, especially in the context of novel social virtual environments such as the Metaverse.

### 2.3 Current state of the literature

All these demonstrations are a testimony to the robustness of the effect and its applicability to a wide variety of contexts. Through these examples, it was shown that the Proteus effect could affect the user's cognitive performance (Banakou et al., 2018), affective state (Peña et al., 2009), motor behavior (Reinhard et al., 2020), physiological responses (Frisanco et al., 2022), or psychological dimensions such as confidence (Yee and Bailenson, 2007) or creativity (Guegan et al., 2016; Gorisse et al., 2023). Two recent meta-analyses corroborated the validity of the effect by finding a small to medium mean effect size (r = 0.24) for the Proteus effect (Ratan et al., 2020; Beyea et al., 2023), an effect size comparable to similar media-related effects. Thus, based on this growing body of literature, it seems reasonable to assume that avatar appearance can indeed affect the behavior, cognition, or emotional state of the users embodying them.

However, if a significant number of studies have been able to replicate the effect, the social, affective, and cognitive processes underlying such a phenomenon do not seem as clear. Indeed, most publications on the subject have focused on demonstrating the effect but very few have sought to explore the theoretical explanations of this effect beyond those given by the first publications about the Proteus effect. Although a few recent reviews of the Proteus effect have highlighted this gap in the literature, and sought to address parts of it (Praetorius and Grlich, 2021; Szolin et al., 2022), these publications did not discuss some of the fundamental shortcomings of the current explanations of the Proteus effect. Indeed, in their systematic review, Szolin et al. (2022) chose to focus on the Proteus effect in the context of video games. They did so in order to study the phenomenon from a more ecologically valid perspective compared to studies using avatars and virtual environments specifically designed for experimental purposes. In that context, the researchers reviewed which avatar characteristics has been found to affect users' behaviors and attitudes while playing video games (e.g., avatar gender, attractiveness or weight) but also how avatars could influence the players' post-game beliefs. This review provided a clear overview of how the Proteus effect can affect players of video games but did not address nor challenge the theoretical understanding of the effect. The review by Praetorius and Grlich (2021) did however discuss this point. The authors conducted a qualitative analysis of the Proteus effect literature in order to classify the selected studies according to a model of identification processes (Looy et al., 2012). This analysis is based on a previous meta-analysis on the Proteus effect (Ratan et al., 2020) that links the Proteus effect to processes of self-identification between the user and the avatar. Although studying the link between identification and the Proteus effect has offered new insights into the mechanisms underlying the effect, Praetorius and Grlich (2021) still believe that the Proteus effect is best explained by the two most widely cited hypotheses on the subject: self-perception theory and priming. Indeed, in their review, both identification and embodiment processes are presented as moderating factors of the Proteus effect, and priming and self-perception processes as the potential underlying causes of the effect. Therefore our review aims to go beyond these previous publications by challenging the widely accepted explanations of the effect as well as proposing new theoretical frameworks to better understand it.

Thus, to inform the theoretical framework of the Proteus effect and avatar embodiment we will confront six different theoretical hypotheses within this review. We will start by addressing the three hypotheses that have already been cited in the Proteus effect literature and their limitations, including self-perception theory, priming, and deindividuation. In the following Section, we will introduce three new hypotheses that may be relevant to better understanding the processes underlying the Proteus effect: cognitive dissonance, embodiment, and perspective-taking. Finally, we will compare all six hypotheses to determine which ones provide a better explanation of the phenomenon.

## 3 Current theoretical hypotheses behind the Proteus effect

In Yee and Bailenson (2007)'s first article on the Proteus effect, the authors cited two existing theoretical frameworks to explain the phenomenon: Self-Perception Theory (SPT, Bem, 1972) and the Social Identity model of Deindividuation Effects (SIDE, Spears and Lea, 1992, 1994; Reicher et al., 1995).

### 3.1 H1: self-perception theory

As the first theoretical hypothesis to have been used to explain the Proteus effect, SPT remains the most cited explanation of the phenomenon to date. SPT states that, in situations where one's internal states (attitudes, emotions, cognition, etc.) are unclear, one might infer them from the external cues they exhibit (i.e., behaviors, circumstances in which a behavior occurs, etc.) (Bem, 1972). More specifically, SPT relies on two main propositions:

1. “Individuals come to ‘know' their own attitudes, emotions, and other internal states partially by inferring them from observations of their own overt behavior and/or the circumstances in which this behavior occurs.”
2. “Thus, to the extent that internal cues are weak, ambiguous, or uninterpretable, the individual is functionally in the same position as an outside observer, an observer who must necessarily rely upon those same external cues to infer the individual's inner states.” (Bem, 1972, p. 2).

According to SPT, if attitude A and behavior/external cue B are commonly associated with each other, when a person exhibits B, they might infer that they must also hold attitude A, in the same way that an outside observer would. Therefore, in the context of the Proteus effect, the theory would suggest that, since the appearance of the embodied avatar is associated with certain attitudes (e.g., aggression, confidence), the user assumes that they must also hold those attitudes.

In Yee and Bailenson (2007) experiment, SPT would posit that, because people tend to perceive taller individuals as more likely to be leaders and competent (Hamstra, 2014), a participant embodying a taller avatar would adopt attitudes commonly associated with these traits (confidence, dominance, etc.). This would cause the participants to be more self-serving during the negotiation task, as it would be congruent with the expectations placed on tall individuals. Likewise, in the case of Banakou et al. (2018), SPT would consider that users perceive Albert Einstein as a figure of intelligence. Therefore, when they see themselves embodying the famous physicist, they must put more effort into the cognitive task to perform well. This allows users to maintain a coherent image of the smart scientist.

### 3.2 H2: deindividuation

Yee and Bailenson (2007) also theorized that the use of immersive virtual reality and avatars could amplify the effect of SPT through the process of deindividuation. According to the most recent model on the subject, deindividuation refers to a psychological state in which a person's behavior is driven by situational norms instead of personal ones (Reicher et al., 1995). Such a state can lead a person to display anti-normative behaviors they would ordinarily never engage in. It has been used to explain extreme behaviors within crowds such as the ones seen in groups of football hooligans for example. It is theorized that deindividuation can be induced by a combination of circumstances such as anonymity, lack of accountability, or group immersion (Vilanova et al., 2017). According to Yee and Bailenson (2007), immersive virtual reality and avatar embodiment could also lead to a similar state.

The study of deindividuation dates back to Le Bon (1895) and his book “The Crowd: A Study of the Popular Mind” where he describes the way people's behavior changes when they are part of a group. Already at that time, Le Bon described how being in a crowd led people to ignore their personal judgment in favor of the group's dynamics. The term deindividuation was later coined by Festinger et al. (1952) and has been extensively researched since in the field of social psychology (for a review see Vilanova et al., 2017). One particular study by Johnson and Downing (1979) demonstrated that when participants were wearing either a nurse's outfit or a Ku Klux Klan robe while administrating fake electrical shocks to a confederate, the participants in the nurse outfit would administer less intense shocks compared to subjects in the KKK robes. Interestingly, they found this effect to be amplified when the participants were deindividuated, resulting in an increase in prosocial behavior when situational cues (i.e., the nurse's outfit) prompted participants to behave that way.

This focus on situational norms was the basis for the most recent theory of deindividuation, the Social Identity Model of deindividuation Effect (SIDE) (Spears and Lea, 1994; Reicher et al., 1995). This model argues that when individuals find themselves deindividuated (e.g., anonymity, group immersion, etc.), they will tend to conform to the norms evoked by the situation even if those norms contradict their personal beliefs. It is believed that this happens as individuals' attention is taken away from their personal characteristics, toward situational norms.

Interestingly, this model has been used to explain some anti-normative behaviors observed in online spaces. Indeed, computer-mediated communication might be prone to induce deindividuation because of factors such as anonymity, distance, or a lack of accountability. For instance, one study found that anonymous players of an online game were more likely to cheat (Chen and Wu, 2015). This link between deindividuation and technologies has led (Yee and Bailenson, 2007) to hypothesize that deindividuation could also occur when individuals are immersed within a virtual environment and embody an avatar. According to this view, this happens when users' attention is diverted away from their personal characteristics and toward the avatar's identity cues. The users then adhere to the social identity evoked by the avatar and conform their behavior and attitudes to it. They hypothesized that deindividuated users would be more likely to follow the norms evoked by their avatar in the same way that the participants in the nurse's outfit did when administering electric shocks (Johnson and Downing, 1979). More specifically, they stated, “Users who are deindividuated in online environments may adhere to a new identity that is inferred from their avatars” (Yee and Bailenson, 2007, p. 274). Overall, the authors concluded that, combined, both deindividuation and self-perception are responsible for the Proteus effect.

### 3.3 H3: priming

Following the introduction of the Proteus effect, Peña et al. (2009) published a study in which they challenged Yee and Bailenson (2007)'s theoretical explanation of the phenomenon. According to them, the Proteus effect could be explained, not through SPT and deindividuation, but simply through priming mechanisms. More specifically, the authors base their claims on the automaticity of social interactions model (Bargh et al., 1996; Bargh, 2006), which argues that there exist automatic perception-behavior links such that perceiving certain stereotypes or associations primes the observer to display matching behaviors or cognitive responses. The authors claimed that, in the context of the Proteus effect, the avatar merely acts as a prime that activates related concepts (e.g., tall avatar activates confidence, leadership, assertiveness, etc.) and inhibits conflicting concepts (e.g., shyness). The activation and inhibition of these concepts then make it more likely for individuals to display behaviors congruent with the prime.

To support their hypothesis, the authors designed two experiments in which they investigated the effects of avatar uniforms on the user, using a desktop computer to display the virtual environment. The first experiment demonstrated that, compared to participants embodying avatars wearing white cloaks, those using avatars wearing black cloaks among a small group of players had more aggressive intentions toward other players and lower group cohesion. In the second experiment, when asked to interpret ambiguous images, participants using avatars with Ku Klux Klan robes were more likely to write stories with higher levels of aggression compared to a control avatar, and showed lower affiliation compared to a doctor avatar.

From these results, the authors concluded that the negatively connoted avatars (i.e., black cloaks and KKK robes) unconsciously primed negative beliefs within the users, which resulted in antisocial intentions and aggressive thoughts. Although these results do not directly contradict the self-perception and deindividuation hypotheses of Yee and Bailenson (2007), the authors argue that priming mechanisms represent a more parsimonious explanation of the Proteus effect. Furthermore, they claim that SPT does not explain the inhibition of conflicting concepts shown in these experiments (e.g., reduced group cohesion and affiliation).

In response to these claims, Yee and Bailenson (2009) published a follow-up experiment arguing that there is a difference between perceiving a concept and embodying an avatar relating to that same concept. Their experiment sought to differentiate possible priming mechanisms from self-perception ones by showing that observing vs. embodying an attractive person did not have the same effects on users. Similarly to their original study, participants either embodied an attractive or unattractive avatar inside the immersive virtual environment. However, at the start of this experiment, people could either observe their avatar in a virtual mirror (i.e., mirror condition) or watch a video of their avatar moving, (i.e., playback condition). Afterward, when asked to choose potential dating partners from a pool of pictures, participants using attractive avatars tended to select more attractive partners than those using unattractive avatars. However, this was only the case for participants in the mirror condition; no difference was observed for the playback condition.

These results demonstrate the importance of avatar embodiment for the Proteus effect to occur and differentiate it from the hypothesized priming mechanisms. The main difference between priming and SPT to explain the Proteus effect is that priming does not distinguish the appearance of the avatar from any other external visual stimulus that might have a priming effect on the user. From the perspective of SPT, however, the avatar is perceived as the physical representation of the user, therefore differentiating it from other visual elements perceived and increasing its relevance to the user. This is what, in their view, made the behavioral change observed in their experiment more powerful.

Finally, a few years later, Ratan and Dawson (2016) proposed an alternative view of the issue that combined both self-perception and priming. In their view, the perception of the avatar triggers the activation of concepts related to the avatar (i.e., stereotypes) in a similar way that priming mechanisms would. Through avatar embodiment, these concepts may become associated with concepts relating to the self. If that is the case, a person's own behavior starts to be influenced by these avatar-related concepts, as seen through the Proteus effect.

### 3.4 Limitations of the current theoretical frameworks

Since the self-perception vs. priming debate, very few studies about the Proteus effect have sought to refine or provide alternative theoretical explanations. Instead, most have focused on demonstrating the effect in a novel experimental context.

In today's literature, most research stands behind hypothesis H1 of self-perception theory as the explanation of the phenomenon based on the original article from Yee and Bailenson (2007). However, SPT also raises a few issues. The first one is that what is considered as “external cues,” from which attitudes are derived, remains rather vague. Indeed, Bem defines these cues as “their own overt behavior and/or the circumstances in which this behavior occurs” (Bem, 1972, p. 2). This explanation applies rather well to situations in which bodily reactions are experimentally manipulated to cause a change in attitude. For instance, hearing a fake accelerated heartbeat while viewing a picture of a woman made men believe they were more attracted to the woman in the picture (Valins, 1966). However, in the case of the Proteus effect, the external cue in question is the appearance of the avatar the user embodies. From Bem's own wording, it can seem farfetched to consider avatar appearance as such an external cue since being tall or attractive, for example, is not an overt behavior. Even by considering the second description of external cues as “the circumstances in which this behavior occurs,” it is hard to describe what “behavior” it could refer to in the context of the Proteus effect. Indeed, in most of the literature, it is simply the sight of the avatar that induces a change in attitude or behavior, not the sight of the avatar displaying a specific behavior.

The second issue with considering SPT to explain the Proteus effect is that it seems quite difficult to falsify experimentally. Indeed, if we do consider avatar appearance to be a valid “external cue” for self-perception, it becomes challenging to test whether self-perception truly explains the behavior change and not an alternative cognitive process. The theory simply tells us that the perception of the avatar's appearance would cause individuals to display an attitude that would be considered congruent with the avatar's image. As highlighted by the priming vs. self-perception debate, self-perception does imply that the avatar has to be perceived as part of the “self,” separated from other cues present in the environment. This subtlety does distinguish possible self-perception processes from priming within the Proteus effect, which was demonstrated experimentally. However, experimentally distinguishing self-perception from other alternative explanations is more challenging.

Perhaps, one of the results that might indicate whether self-perception processes are involved in the Proteus effect comes from a study in which participants embodied either creative-looking avatars or control avatars (Buisine and Guegan, 2019). This experiment found, among other results, that using creative-looking avatars increased participants' creativity during a brainstorming task. In addition, researchers also measured participants' perception of the creative identity of the avatars. They tested whether that variable would mediate the relationship between the type of avatar and their creativity during the task. They found that the creative-looking avatars were indeed perceived as more creative than the control avatars. However, that perception did not mediate the relationship between avatar appearance and creativity. This result indicates that consciously identifying the avatar as more or less creative did not influence the fact that the avatar could affect the participant's creativity. In the context of self-perception, it can be argued that one would have to perceive their avatar as creative for the effect to work, which is not the case here. This example does not constitute an actual demonstration that self-perception processes cannot explain the Proteus effect (e.g., the perception of the avatar's creative image could happen unconsciously); however, investigating these subtleties will bring more clarity to this question.

Another aspect of SPT that could be tested comes from the notion that, according to the theory, the person's internal states must be somewhat “weak, ambiguous, or uninterpretable” for self-perception to occur. This then results in the person having to rely on external cues to deduce their attitudes. Thus, investigating whether individuals' internal states really are inaccessible in the context of a Proteus effect protocol would provide more evidence to understand if self-perception might explain the effect.

Overall, beyond its shortcomings, SPT remains a solid theory to explain the Proteus effect. For instance, compared to other frameworks, it highlights the importance of the self and avatar embodiment within this effect as highlighted by Yee and Bailenson (2009).

The second hypothesis (H2) that was brought up was the role of a potential deindividuated state within the users of virtual environments that may explain why they adhere to the identity of their virtual representation. Even though Yee and Bailenson (2007) only considered deindividuation in combination with self-perception processes to explain the effect, deindividuation could also be considered as an explanation of the effect on its own. Indeed, in the same way that a deindividuated person immersed in an aggressive crowd may become aggressive, the user embodying an avatar inside a virtual environment could also be inclined to match their attitude and behavior to the identity of the avatar. This hypothesis would also explain why virtual reality and avatars have successfully shaped behavior in a way that behavioral priming has not, as demonstrated by the failed attempts at reproducing Bargh et al. (1996)'s experiment (Doyen et al., 2012) which was later successfully reproduced using the Proteus effect (Reinhard et al., 2020). In addition, deindividuation potentially emphasizing the Proteus effect could also partly explain why a recent meta-analysis has found that immersive virtual reality setups seem to drive a stronger Proteus effect compared to PC screen displays (Beyea et al., 2023). Indeed, the virtual reality setup would provide a higher level of deindividuation as the user's body is replaced by the avatar's, concealing crucial identity cues. However, no experiments so far have explicitly tested the hypothesized role of deindividuation in the Proteus effect. To do so, future studies could aim to experimentally shift the user's focus away from the situational norms evoked by the avatar to instead redirect it toward their own personal norms. This could be achieved by increasing the salience of the user's identity within the virtual environment, as was done in past deindividuation protocols (Festinger et al., 1952).

Finally, regarding the priming hypothesis (H3), its flaw was well expressed in the title of Yee and Bailenson (2009)'s paper “The Difference Between Being and Seeing.” However, this does not mean that the cognitive mechanisms ascribed to priming are completely unrelated to the mechanisms underlying the Proteus effect. Indeed, some priming processes could explain how the visual perception of the avatar can activate a semantic network of concepts relating to the appearance of the avatar (i.e., spreading activation, Anderson, 1983). These mechanisms have been used to explain the results of classical priming experiments such as word completion tasks. Furthermore, in the context of the Proteus effect, it is difficult to explain how the perception of an avatar could lead to the activation of related concepts without a process such as spreading action. However, the fact that concepts become more salient in memory does not explain why participants would go as far as changing their behavior or attitudes after this activation of semantic networks.

A specific sub-field of priming research actually relates to the Proteus effect: behavioral priming. This type of priming was mostly investigated between 1990 and 2010 and provided some very well-known results. For example, one of these studies infamously showed that simply priming participants with words related to intelligence led them to have higher scores on a general knowledge test (Dijksterhuis and van Knippenberg, 1998). Another similar paper related that priming participants with words associated with elderly people made them walk slower when exiting the experiment (Bargh et al., 1996). The theory behind these papers was that priming individuals with a specific concept could unconsciously elicit behaviors related to that concept. Unfortunately, this specific field has suffered from the replication crisis, as many subsequent studies were unable to replicate the results of both experiments cited above. Specifically, a recent study was only able to replicate the results of Bargh et al. (1996) when the experimenter charged with measuring the pace of the participants was prompted to believe participants would indeed walk slower (Doyen et al., 2012). This indicates that the original results were probably simply caused by confirmation bias since the experiment was most likely not a double-blind protocol. Those results do not indicate that behavioral priming is entirely false as a whole, but it shows that the effect, if it exists, is probably smaller and less common than originally assumed.

If today this framework lacks credibility, it is interesting to note that the Proteus effect has been used successfully to demonstrate very similar results with avatar embodiment. Indeed, as cited earlier, Reinhard et al. (2020) showed that embodying an elderly avatar led participants to walk slower after exiting the virtual environment. Unlike the original experiment, this one followed a strict double-blind protocol, adding to the credibility of the results.

Therefore, it seems that trying to evoke a certain behavior through a word prime has a less robust effect than inducing said behavior through the embodiment of a specific avatar. The main identifiable difference between those two protocols seems to reside in how the primed concept relates to the subject. Indeed, relating a concept to the self by way of avatar embodiment seems to be more efficient at evoking a congruent behavior in the user than the mere presentation of words referencing that same meaning. This idea actually echoes previous findings that showed that the effects of a prime could be amplified when self-relevance to the prime was higher. For example, a study on stereotype threat showed that black participants performed worse on a cognitive test after reading a text about a black person from a first-person perspective than they did when reading it from a third-person perspective (Marx and Stapel, 2006). Overall, hypothesis H3 cannot explain the Proteus effect on its own. However, processes relating to priming such as spreading activation might still be involved in the effect.

## 4 Further theoretical hypotheses

In this Section, we will present three additional hypotheses that may provide more insight into the theoretical understanding of the Proteus effect: cognitive dissonance, avatar embodiment, and perspective-taking.

### 4.1 H4: cognitive dissonance

Interestingly, SPT was originally developed as an alternative explanation to the theory of cognitive dissonance (Festinger, 1957). Cognitive dissonance is thought to be a state of psychological stress people experience when their actions contradict their beliefs (e.g., being concerned for animal welfare but eating meat) (Festinger, 1957). It is theorized that people try to minimize this dissonance when it occurs by either changing their behavior or their beliefs (e.g., stopping eating meat or rationalizing animal suffering).

The difference between SPT and cognitive dissonance is that in SPT, people do not experience this state of psychological distress in response to contradictions. Instead, people's attitudes are simply shaped to be congruent with their own behavior. Even though these two frameworks were introduced as competing theories, it was later accepted that each explained slightly different phenomena (Fazio et al., 1977). It is thought that SPT is better suited to describe situations in which people's internal states are relatively ambiguous or vague whereas cognitive dissonance applies to situations in which people clearly display a strong contradiction between their beliefs and actions. Today, SPT is mainly cited within areas such as marketing and persuasion (e.g., the foot-in-the-door technique, Burger, 1999) whereas cognitive dissonance remains a highly influential theory across many domains of psychology (Harmon-Jones et al., 2015; Kaaronen, 2018).

More generally, however, one could argue that, at its core, what SPT and cognitive dissonance try to describe is a deeper intrinsic need for humans to try to remain coherent. Both theories illustrate people's drive to have congruent attitudes/internal states and behaviors/external cues and vice versa.

Interestingly, this description of the phenomenon echoes recent work on predictive processing to explain brain functioning and behavior (Friston, 2009). Put simply, the theory of predictive processing (or predictive coding) posits that the brain constructs a generative model of its environment based on prior knowledge (Clark, 2013; Millidge et al., 2021). This representation is then used to make predictions about upcoming sensory events, which, in turn, are compared to the actual upcoming sensory information. Any prediction errors are then used to update the generative model of the environment to minimize future errors. In a recent article, Kaaronen (2018) proposed a new model of cognitive dissonance based on predictive processing: the theory of predictive dissonance. As the author explains, both frameworks fundamentally rely on the idea that individuals aim to reduce surprise (i.e., dissonance or prediction errors). Furthermore, bringing elements of predictive coding within cognitive dissonance theory allows us to explain certain issues related to the cognitive dissonance theory.

This new account of cognitive dissonance shows how this theory originally drafted in Festinger (1957) remains highly relevant in today's literature and even relates to some of today's most influential theories of brain functioning. All of this highlights how fundamental this drive for consistency is for individuals, which is also illustrated in SPT. Interestingly, some findings attributed to SPT could also be included within this wider framework such as the fact that inducing specific facial expressions can shape people's subsequent attitudes (Laird, 1974; Ito et al., 2006). This could very well be explained in terms of prediction errors of upcoming sensory information causing, down the line, a change in attitude.

Thus, we could try to understand the Proteus effect in terms of minimizing the dissonance created by an attitude that would be incongruent with the perceived appearance of the avatar. In other words, the user embodying a tall avatar takes on an assertive attitude to avoid being incoherent regarding the image they have of tall individuals. This account could provide an alternative explanation to SPT that still addresses this drive for coherence between internal and external states without the shortcomings associated to SPT's definition. Furthermore, this theoretical framework is linked to a large and contemporary body of literature within the cognitive sciences, giving the phenomenon more context.

### 4.2 H5: embodiment

As mentioned in previous hypotheses, avatar embodiment plays an important role in the Proteus effect. However, while most definitions of the effect will mention “avatar embodiment,” not all studies actually define the notion of embodiment and what role it could play in the behavioral change observed.

The sense of embodiment in virtual reality is defined by Kilteni et al. (2012) as a combination of three different factors: a sense of agency (SoA), a sense of self-location (SoSL), and a sense of body ownership (SoBO). The SoA describes the feeling of having motor control over the body and being aware of its movements. In virtual reality, visuomotor synchronicity between the user's and the virtual body's movements is crucial to instill a SoA within the user. SoSL refers to the feeling of being located in a space, which is typically our own physical body but can also apply to an avatar in a virtual environment. This can change even more in out-of-body experiences where individuals report feeling located outside their own bodies (Ehrsson, 2007; Lenggenhager et al., 2012). Finally, SoBO is defined as recognizing a body as our own. This is the sense that is manipulated in the famous rubber hand illusion in which participants felt ownership over a plastic hand located close to their real hand (Botvinick and Cohen, 1998). This was done by visually hiding the participant's real hand under a sheet and the use of synchronous tactile stimulation of both the real and the fake hand. Each of these three factors have been experimentally manipulated within virtual environments to understand how an optimal level of embodiment could be felt by users. Fribourg et al. (2020) did so by using the user's level of control over the avatar to influence the SoA, the avatar's appearance (i.e., minimal or realistic) to change the SoBO and the user's point of view (i.e., first or third person) to alter the SoBL.

Regarding the Proteus effect, multiple studies have sought to understand how these different aspects of embodiment may shape the effect. Several of these studies have found that a higher sense of embodiment was linked to a stronger Proteus effect (Kilteni et al., 2013; Beaudoin et al., 2020; Frisanco et al., 2022). More specifically, two of these studies (Kilteni et al., 2013; Beaudoin et al., 2020), found the SoBO to be associated with a higher Proteus effect. This last finding is coherent with literature about the SoBO as it has been shown that multiple aspects of avatar appearance such as realism or similarity can modulate SoBO (Argelaguet et al., 2016; Lin and Jörg, 2016). These results seem to indicate a possible moderating effect of embodiment on the Proteus effect and even point to SoBO as the underlying process. However, several other studies did not find such a link between embodiment and the Proteus effect (Ratan and Sah, 2015; Verhulst et al., 2018; Reinhard et al., 2020), thus contradicting the previously cited results.

Based on these conflicting accounts, a recent study aimed to test whether the sense of embodiment does in fact moderate the Proteus effect (Dupraz et al., 2024). Interestingly, their results revealed that participants' embodiment levels did not impact the Proteus effect, therefore refuting the notion of a moderating effect. Based on these results, the authors hypothesized that despite lower embodiment levels in certain experimental conditions, the participants still established a link between the avatar and themselves, allowing for the Proteus effect to occur regardless. It could be theorized that this hypothesized link between the self and the avatar could be explained by identification processes also mentioned in this article. Indeed, such processes are defined as the experience of assimilating some of the avatar's characteristics into the user's self-perception (Klimmt et al., 2009). These have previously been presented as potential influences of the Proteus effect (Ratan et al., 2020; Praetorius and Grlich, 2021), however, to our knowledge, no study has currently tested the influence of identification processes within the Proteus effect.

Future studies could therefore aim to test such a hypothesis. If identification processes do indeed play a role within the Proteus effect, they could potentially explain why previous studies have found conflicting results when testing the moderating effect of embodiment on the Proteus effect (Dupraz et al., 2024). Indeed, it could be theorized that high levels of identification could compensate for lower levels of embodiment and still facilitate the Proteus effect. The interaction between these two processes should therefore be tested in future studies.

All in all, current research about the role of embodiment within the Proteus seems inconsistent. The disparity in the literature could potentially be explained by additional underlying processes at play during avatar embodiment that are not being measured as of now. Therefore, broadening our understanding of the processes involved in the Proteus effect could help clarify how embodiment fits within this framework.

### 4.3 H6: perspective-taking

The final hypothesis surrounding the effects of avatar embodiment that will be addressed comes from a slightly different side of the literature than the Proteus effect. Some researchers have used immersive virtual reality and avatars to induce perspective-taking experiences that aim to reduce negative biases among users. Within this literature, researchers hypothesize that embodying an avatar representing a person from a marginalized group (e.g., the elderly, women, certain ethnicities, etc.) will increase the user's empathy for the group in question (Loon et al., 2018). This then prompts the users to display behaviors that go against the negative stereotypes associated with the avatar.

Interestingly, based on this description, the framework generally makes opposite predictions to the Proteus effect (Clark, 2020). Indeed, the Proteus effect assumes that embodying an avatar will cause the user to adopt behaviors that would be coherent with any stereotypes associated with the avatar's appearance. On the contrary, virtual reality perspective-taking predicts that users will not display stereotypical behaviors, as their empathy toward the avatar has increased through the embodiment.

Both sides of the literature include experiments supporting each opposing view. Some studies using virtual reality perspective-taking have shown that embodying black avatars led participants to show a decrease in implicit racial bias (Peck et al., 2013). Another showed similar results on implicit ageism using elderly avatars (Oh et al., 2016). On the other hand, Proteus effect research has shown that embodying the avatar of an individual from a stereotyped group has led participants to display behaviors and attitudes in line with the stereotypes. For instance, Pea et al. (2016) demonstrated that participants using avatars with a larger body showed a decrease in physical activity compared to people using thinner avatars. In this experiment, participants displayed behaviors that were coherent with negative stereotypes associated with larger individuals (i.e., being lazy). Similar results were found regarding negative racial stereotypes (Ash, 2016).

This nuance between increasing empathy toward a stigmatized group and accentuating negative stereotypes toward said group through avatar embodiment highlights the need for a better understanding of the Proteus effect. The distinction between these two views may lie in the framing of the embodiment experience. For instance, in their experiment assessing how perspective-taking could impact negative biases toward elderly people, Oh et al. (2016) specifically asked the participants to “Imagine a day in the life of this individual, looking at the world through her/his eyes and walking through the world in her/his shoes” (p.402) while embodying an elderly avatar. This emphasis on placing oneself in the avatar's perspective is not common in Proteus effect studies and would be more likely to promote empathy. In addition, the perspective-taking approach only applies to experiments using avatars of marginalized groups (e.g., gender, race, sexuality, etc.). Even though it can be argued that the Proteus effect does rely on having more or less accurate preexisting stereotypes about whom the avatar represents (e.g., engineers are creative), those are not necessarily negative and would not foster empathy in the user embodying them. Thus, this framework does not apply to a large part of the Proteus effect literature that uses avatars unrelated to those social issues (e.g., alien avatars, engineers, Einstein, tall individuals, etc.). Overall, the perspective-taking hypothesis cannot account for many of the results found within the Proteus effect literature and seems to account for a different phenomenon associated with avatar embodiment.

## 5 Discussion

This review aimed to provide a critical analysis of different theoretical frameworks that may explain the underlying processes of the Proteus effect. A total of six different hypotheses were assessed through this review. As of now, there does not seem to be a unique hypothesis that can explain the effect all by itself, but rather a combination of a few hypotheses that may explain different aspects of the phenomenon. All six hypotheses are summarized in Table 1. For each hypothesis, its mechanisms are described along with how this framework would apply to a specific example of the Proteus effect. Whenever applicable, we used the protocol of Banakou et al. (2018) as an example.

**Table 1:** Summary of the six hypotheses aiming to explain the underlying processes of the Proteus effect, their mechanisms, and examples.

**Hypotheses**	**Mechanisms**	**Example**
H1: Self-Perception Theory	Attitudes of the user are derived from observing external cues (avatar appearance) and match what an outside observer would find coherent.	The user embodies Albert Einstein. An external observer would expect Einstein to perform well in a cognitive task. Therefore, the user puts more effort into the task to perform well.
H2: Deindividuation	The use of virtual environments and avatar embodiment deindividuates the user. They then conform to the situational norms evoked by the avatar's appearance.	The user embodies Albert Einstein. This experience shifts the user's attention from their personal norms toward the norms evoked by Einstein. They then conform to those norms and perform better on the cognitive task.
H3: Priming	The sight of the avatar activates networks of concepts associated with the avatar's appearance. This prompts the user to display behaviors related to these concepts.	The user embodies Albert Einstein. Viewing the avatar activates concepts related to intelligence within the user, which prompts them to act accordingly and perform well on the cognitive task.
H4: Cognitive dissonance	The user aims to keep their behavior and attitudes coherent with what the appearance of their avatar suggests to avoid feeling dissonance.	The user embodies Albert Einstein. Since embodying a figure related to intelligence and performing poorly on a cognitive task would be incoherent and create dissonance, the user puts in more effort to perform well.
H5: Embodiment	The user's subjective experience of embodying the virtual body of the avatar and processing it as it was their own body.	The user embodies Albert Einstein. Through a high sense of agency of over the movements of the avatar, ownership over the virtual body and feeling located inside the avatar, the user processes the virtual body as its own which facilitates the emergence of the Proteus effect.
H6: Perspective- taking	The experience of embodying an avatar of a different individual prompts the user to feel empathy toward them. The user then adopts attitudes that oppose any negative stereotypes associated with that individual.	The user embodies the avatar of a woman. This experience increases the user's empathy toward women. Therefore, when confronted with a mathematics test, the user puts more effort into the task to contradict negative stereotypes about women.

Thus, to summarize, the self-perception hypothesis (H1) conveys that the Proteus effect relies on a need for coherence between external cues and attitudes. In addition, it also emphasizes the importance of embodying the avatar for the Proteus effect to occur (Yee and Bailenson, 2009), however, this hypothesis has a few limitations. As discussed previously, whether this theory can be applied to the Proteus effect remains unclear and should be further tested in upcoming research. Furthermore, beyond its limitations, the strong points of the theory cited earlier can be found in alternative hypotheses such as cognitive dissonance or avatar embodiment. Therefore, even if we do not claim that it is impossible for SPT to explain the Proteus effect, we propose an alternative view of the processes responsible for the effect, described at the end of this Section, that does not include SPT and reject H1.

On the other hand, deindividuation (H2) could represent an interesting explanation of the phenomenon and more specifically, why virtual reality and avatar embodiment are powerful means of inducing specific behaviors within users. Furthermore, this framework is linked to previous research that provides additional context to the Proteus effect, notably the effect of certain uniforms or costumes on behavior (Diener et al., 1976; Johnson and Downing, 1979; Greco, 2019). This hypothesis remains to be tested but provides a strong explanation of the effects of avatar embodiment on behavior from a social psychology point of view. Based on this we choose to include H2 in our framework.

As for the priming hypothesis (H3), as explained, it does not account for the importance of avatar embodiment. However, the cognitive mechanisms associated with priming (i.e., spreading activation) could explain how networks of related concepts are activated after the perception of an avatar and thus, how individuals can go from concepts such as “tall” to “confidence.” Nonetheless, we reject H3 since priming mechanisms as a whole do not seem to explain the Proteus effect.

H4 brought forward the idea of cognitive dissonance as an alternative explanation to SPT. This allows us to understand the Proteus effect through the lens of trying to minimize dissonance potentially caused by a discrepancy between one's appearance (the avatar) and one's behavior or attitude. This hypothesis also fits within a wider framework that aims to explain such processes as a global need for coherence between internal processes and our behavior. Therefore, we also include H4 in our framework.

Additionally, having a better understanding of how avatar embodiment (H5), and its sub-components, play a role in the Proteus effect would provide crucial details about the underlying mechanisms of the effect. Preliminary research on the matter does seem to indicate that the sense of embodiment of the user inside the virtual environment could influence the strength of the effect. Therefore, future research should aim to deepen our understanding of this interplay between embodiment and Proteus effect. Nevertheless, we include H5 within our framework.

The last hypothesis (H6) focused on the view that avatar embodiment could induce a perspective-taking experience in the user by seeing the world through the eyes of someone else. Even if perspective-taking studies have demonstrated that it is possible to increase empathy for the avatar through the use of virtual reality, these studies use different methods that are specifically aimed at inducing such feelings in the user. We choose to reject H6 as it cannot explain all the results found within the Proteus effect literature since they often contradict the predictions made by the perspective-taking framework.

All in all, we propose an alternative view of the underlying mechanisms of the Proteus effect. This view includes the idea that the use of immersive virtual reality, virtual environments, and avatars induces a sense of deindividuation within the user. This state makes the user more likely to conform to the behavioral and attitudinal expectations evoked by the appearance of the avatar. In addition, we also hypothesize that this behavioral and/or attitudinal change is also encouraged by a drive to minimize any possible dissonance felt when the avatar's appearance is incongruent with the behavior of the user. Finally, this framework also takes into account the sense of embodiment experienced by the user while embodying the avatar. Based on previous research we expect that a stronger sense of embodiment would result in a stronger Proteus effect.

It is important to note that the notions of deindividuation or cognitive dissonance have never been tested in the context of the Proteus effect and therefore remain rather hypothetical. Future research should aim to refine our understanding of these processes. For example, this can be done by systematically measuring the sense of embodiment of users, testing whether deindividuation or self-perception processes are at play in the effect, or even differentiating the processes at play in perspective-taking experiences from the Proteus effect.

## 6 Conclusions

Overall, this review highlights the need for a better theoretical understanding of this phenomenon and the approaches that could help us do so. Research focused on how immersive technologies and virtual environments affect their users is crucial as these elements become increasingly common. Moreover, studying phenomena like the Proteus effect provides us with new unique insights into human cognition, which would not have been possible without the technology. In addition, this effect has the potential to be applied to a large number of domains in order to improve specific beneficial skills. It has already been shown to work within a professional context to tailor the creativity processes of workers to specific demands (Buisine et al., 2016).

Finally, from a wider perspective, the Proteus effect highlights an interesting human characteristic: our adaptability. From the outside, it can seem rather absurd that simply putting a virtual reality headset on and visualizing a new virtual body can automatically prompt us to act differently. However, this effect could reflect a deeper intrinsic quality that allows us to adapt to new situations such as using novel technologies.

This notion of adaptability is reminiscent of a scientific debate based on Lewin (1936)'s equation staging the person and the situation as fundamental drivers of human behavior. Some researchers claimed that people were not as consistent across situations as some research on personality traits had previously claimed (Mischel, 1968). In this argument, Mischel (1968) believed behavior to be more dependent on the situation than on fixed personality traits. Even though today's arguments are more nuanced about the contribution of each factor on behavior, this debate still highlights people's tendency to be more adaptable in response to different situations than we may think.

From an evolutionary perspective, having this degree of flexibility in response to new situations seems coherent. A good balance between flexibility and consistency would represent an evolutionary advantage. A completely inconsistent person that would change entirely depending on each situation would be too unreliable within a society. Likewise, an inflexible person, always behaving according to the same norms, would struggle to survive whenever their environment inevitably changes. The Proteus effect could therefore be a very modern demonstration of this intrinsic adaptability to new realities.

## Author contributions

AM: Writing – review & editing, Writing – original draft, Investigation, Conceptualization. BB: Writing – review & editing, Writing – original draft. SB: Writing – review & editing, Writing – original draft.
